# *PARP1* Inhibition as a Novel Therapeutic Target for Keloid Disease

**DOI:** 10.1089/wound.2018.0910

**Published:** 2019-05-03

**Authors:** Tae Hwan Park, Chan Woo Kim, Jin Sik Choi, Yun Joo Park, Yosep Chong, Min Ji Park, Yuri Cho

**Affiliations:** ^1^ Department of Plastic and Reconstructive Surgery, CHA Bundang Medical Center, CHA University School of Medicine, Seongnam, Republic of Korea.; ^2^ Department of Radiology, Hallym University Sacred Heart Hospital, Hallym University College of Medicine, Anyang, Republic of Korea.; ^3^ Department of Hospital Pathology, Yeouido St. Mary's Hospital, The Catholic University of Korea College of Medicine, Seoul, Republic of Korea.; ^4^ Department of Internal Medicine, Gangnam CHA Medical Center, CHA University School of Medicine, Seoul, Republic of Korea.

**Keywords:** keloid, *PARP1*, rucaparib, fibroblast

## Abstract

**Objective:** Inactivation of poly(ADP-ribose) polymerase 1 (*PARP1*) has been found to have protective effect in several fibrotic diseases. But the effect is not studied yet in keloids. Herein, we evaluated the therapeutic effect of *PARP1* inhibitor, rucaparib, for keloids.

**Approach:** The protein expressions of *PARP1* and smad3 were evaluated with western blotting in keloids and controls. The effect of rucaparib was evaluated using 3-(4,5-dimethylthiazol-2-yl)-2,5-diphenyltetrazolium bromide assay and migration assay. We further analyzed the effect of rucaparib on patient-derived keloid xenograft murine model.

**Results:** The protein expressions of *PARP1* and smad3 were significantly higher in keloid tissue. Rucaparib (20 μM) significantly suppressed the proliferation of keloid fibroblasts. Moreover, the combination of rucaparib (20 μM) and triamcinolone (50 μM) showed additive suppressive effect on keloid fibroblasts. Migration assay showed that rucaparib (10 μM) significantly suppressed the migration of keloid fibroblasts. Fibrosis markers in keloid fibroblasts significantly decreased after rucaparib treatment (20 μM). In patient-derived keloid xenograft model, rucaparib significantly reduced the size of keloid tissue.

**Innovation and Conclusion:** The study data suggest *PARP1* might be a novel therapeutic target for keloid disease. *PARP1* inhibitor, rucaparib, might be a promising therapeutic drug for the treatment of keloid disease.

**Figure:**
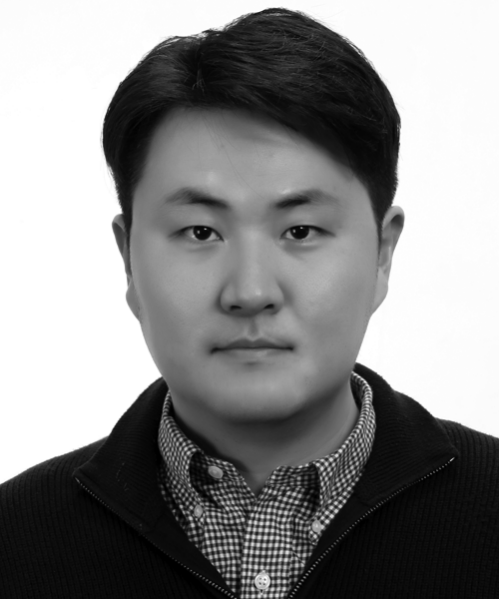
Tae Hwan Park, MD, PhD

**Figure:**
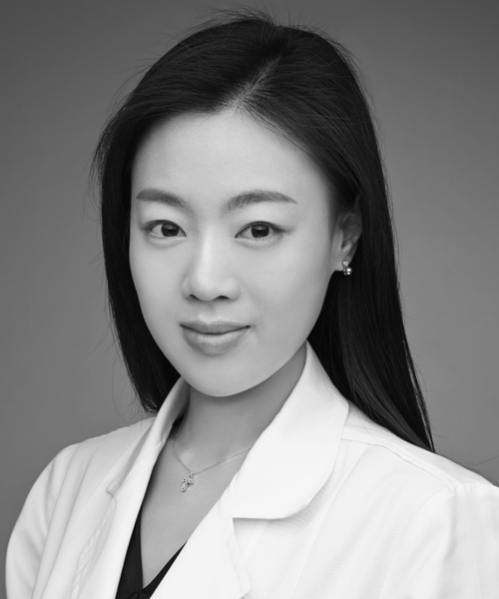
Yuri Cho, MD, PhD

## Introduction

Keloid disease, which is caused by impaired appropriate stop signals in wound healing processes, results in aberrant proliferation of dermal fibroblasts, inflammation, and excessive deposition of extracellular matrix.^1,2^ The term “keloid” was coined in 1806 to describe the crab claw-like appearance of the scar and is strictly defined as scars that spread beyond the boundaries of the original wound and that do not regress spontaneously.^1,3^ In addition to a genetic predisposition, other factors such as skin tension, deregulated wound healing, immune dysfunction, abnormal apoptosis, and sebaceous gland density have been implicated in the etiology of keloid scarring. These pathogenic processes are mediated by complex signal transduction cascades that form crosstalk networks between many different signaling pathways, including the transforming growth factor-beta 1 (TGF-β1)-Smad3 pathway,^4–7^ interleukin-6 (IL-6) pathway,^8–13^ insulin-like growth factor pathway,^14,15^ and mitogen-activated protein kinase pathway.^16,17^

The complexity of the wound-healing process and the lack of proper animal models for keloid scar formation have also restricted progress in research aimed at revealing these mechanisms. However, recent studies validated reliable humanized keloid implantation models using immunocompromised or immunocompetent animals.^18–20^ Considering that immune network between host and microenvironment is an important factor responsible for keloid disease, immunocompromised animal models lacking T cell or B cell immune responses may not recapitulate that of the original keloid immune response. Therefore, we adopted patient-derived keloid xenograft models with immunocompetent murine models to provide a more accurate reflection in this study.^18^

Poly(ADP-ribose) polymerases-1 (*PARP-1*) is the molecule involved in DNA repair, balance of cellular energetic pools, which culminates with necrosis and cell dysfunction, and expression of proinflammatory genes.^21^ In fact, overactivation of *PARP-1* has been found in many physiological conditions, such as inflammatory injury, which are triggered by oxidative stress and DNA damage. Recently, therapeutic usefulness of *PARP-1* has been implicated in several fibrotic conditions, such as liver, kidney, and lung, where *PARP-1* inhibitors attenuate the disease progress and fibrotic detrimental effects.^22–24^

## Clinical Problem Addressed

Over several decades, no single treatment option has been advocated and none of them produced consistent and effective therapeutic results, and high rates of recurrence are common after surgery alone. Many authors have proposed numerous treatment options, but none of them clearly successfully eradicated keloid disease.^25–27^ These findings reflect the current lack of knowledge regarding the exact molecular mechanisms and pathogenic mechanisms that underlie keloid formation. The *PARP1* inhibitor, rucaparib is a recently FDA-approved therapy for ovarian cancer. In the current study, we would like to reveal the *in vitro* effect of pharmacological inhibition with rucaparib in terms of cell migration, proliferation, and expression of fibrosis-related markers. We further analyze its effect *in vivo* using patient-derived keloid xenograft model.

## Materials and Methods

### Ethics approval

Following approval from the Institutional Review Board in CHA Bundang Medical Center, which adheres to the ethical standards as formulated in the Declaration of Helsinki, keloid tissues were obtained from eight patients undergoing surgical excision after obtaining a written informed consent from all of the patients. Keloid diagnosis was made on the basis of its clinical and pathological findings.

### Patients

Patients with keloids who presented to the outpatient clinic were included in the study based on the following criteria: (1) the scar was elevated and extended beyond the dimensions of the initial injury site or lesion; (2) patients were older than 18 years; (3) surgical excision was scheduled; (4) patients received no additional treatment or medication during the study and before surgical excision; and (5) patients signed up for the data use agreement as a basis to the clinical study. Patients were excluded from the study if they were unavailable for follow-up or wanted to stop treatment for any reason. Patients who had received any additional adjuvant therapy during the treatment were also excluded from the study. A total of eight keloids on nine patients were included in this study and all keloids showed deep thickness. The detailed information of the cases is listed in Table 1.

**Table 1.** Baseline demographics

*No. of Cases (n)*	*Age (years)*	*Gender*	*Skin type (Fitzpatrick)*	*Duration of Scar (years)*	*Scar Location*	*Cause*
1	21	F	F III-IV	7	Abdomen	Spontaneous
2	56	F	F I-II	30	Chest wall	Infection
3	21	F	F III-IV	7	Axilla	Spontaneous
4	60	F	F I-II	25	Umbilicus	Surgery
5	37	F	F III-IV	5	Abdomen	Surgery
6	21	F	F I-II	3	Helix	Piercing
7	27	F	F I-II	3	Lobule	Piercing
8	23	F	F I-II	4	Lobule	Piercing
9	15	F	F I-II	2	Helix	Piercing

### Animal care

All animal protocols used in this study were approved by the Institutional Animal Care and Use Committee of CHA University. Four 7-week-old mice (ICR mice) (Orient Bio Co., Seongnam, South Korea) were housed separately in an animal resources facility, at a controlled temperature (20–22.8°C) with a 12-h light/12-h dark cycle. Chow and water were provided *ad libitum*.

### Tissue handling and implantation

We used the patient-derived keloid xenograft mouse model to confirm the *in vivo* efficacy of rucaparib on keloid tissue. Immediately after surgical excision, a plastic surgeon deepithelialized human keloid tissue and evenly cut it into two pieces (1.0 × 1.0 × 1.0 cm^2^) with #11 surgical blade; After immersion into Dulbecco's modified Eagle's medium (DMEM) solution, we implanted deepithelialized human keloid tissue into the ventral subcutaneous pocket of mice of 7 weeks of age, and closed the wound with nylon 5–0. Appropriate dressing was done to minimize wound complications.

### *In vivo* explantation

The animals were randomly assigned into two groups consisting of two mice each, depending on whether they will be injected with *PARP1* inhibitor administration (experiment group), or without *PARP1* inhibitor but with normal saline pretreatment (control group) 1 week afterward.

Seven days afterward, the experiment group (*n* = 2) was then treated with rucaparib (1 mM, 0.5 mL) by subdermal injection just around the implanted site. Meanwhile, the control group (*n* = 2) was treated with 0.5 mL of PBS.

Twelve weeks postoperatively, we harvested the implanted keloid tissue with sterile draping. We measured the dimensions along with documentation, the firmness of the tissue.

### Primary culture of keloid fibroblasts and NHDF

The fibroblasts were isolated from the dermis by surgical excision. The dermis was cut into ∼5 mm^3^ pieces. The epidermis and lipid layer were removed with 2% dispase II (Sigma, St. Louis, MO) and the connective tissue was digested in 0.5 mg/mL collagenase A (Sigma) at 37°C for 3 h using a water bath. The digested solution was filtered through a 70 μm strainer (BD Biosciences, San Diego, CA). The cell pellets were resuspended in, and washed with, 1 × Dulbecco's phosphate-buffered saline (Gibco, Gaithersburg, MD). The cells were cultured in DMEM medium (Gibco) supplemented with 10% fetal bovine serum (FBS, Gibco) and 1% penicillin/streptomycin (Gibco) at 37°C and 5% CO_2_. The medium was replaced every 2–3 days and the cells were subcultured at 70–80% confluency. NHDF was also used as control. All experiments were performed with cells at passage 3.

### Cell proliferation analysis (MTT assay)

With the CellTiter 96 Aqueous One Solution Cell Proliferation Assay (Promega, Madison, WI), cell proliferation was measured on the basis of cellular conversion of the colorimetric reagent 3-(4,5-dimethylthiazol-2-yl)-2,5-diphenyltetrazolium bromide (MTT) into soluble formazan by dehydrogenase enzyme found in metabolically proliferating cell. Following each treatment, 20 μL of dye solution was added into each well in a 96-well plate and incubated for 2 h. Subsequently, the absorbance was recorded at a wavelength of 490 nm using an enzyme-linked immunosorbent assay (ELISA) plate reader (Molecular Devices, Sunnyvale, CA).

### Migration assay

The chemotactic migration of fibroblasts was measured with a transwell migration apparatus. Conditioned medium derived from the culture of keloid or normal fibroblasts was added to the lower wells of the transwell chamber. Fibroblasts were trypsinized, resuspended in serum-free DMEM at a concentration of 1.2 × 10^4^ or 2 × 10^4^ cells/mL, and added into the upper wells of the transwell chamber with a filter with 8-μm pores. The chambers were incubated for 12 h, and the interior of the inserts was gently swabbed to remove nonmigratory cells. Gentle washing of the transwell for 2 min in 1 × PBS was performed. The transwell was placed into a 4% paraformaldehyde solution for 15 min, before washing with 1 × PBS. The insert was transferred to a clean well containing 700 μL of 0.1% Crystal Violet and incubated for 30 min at room temperature. Crystal Violet stain was removed and washed twice by PBS. Each insert was transferred to an empty well, and 200 μL of 100% acetic acid per well was added. Then, 100 μL from each sample was transferred to a 96-well plate and was measured by the OD 560 nm in a plate reader.

### Immunoblot analysis

Cells were lysed for 20 min on ice with lysis buffer and centrifuged at 14,000 *g* for 10 min at 4°C. Samples were resolved by sodium dodecyl sulfate/polyacrylamide gel electrophoresis, transferred to nitrocellulose membranes, blotted with appropriate primary antibodies at a dilution of 1:1000, and treated with peroxidase-conjugated secondary antibodies (Biosource International, Camarillo, CA). We also performed electrophoresis of protein extracts derived from keloid or normal dermal tissue using a Tris-glycine buffer system, and subsequent blottings were performed. Bound antibodies were visualized using chemiluminescent substrate (ECL; Amersham, Arlington Heights, IL) and exposed to Kodak X-OMAT film (Kodak, New Haven, CT). Primary antibody for rabbit anti-*PARP1* was purchased from Cell Signaling Technology (Danvers, MA). Goat anti-actin antibody was from Santa Cruz Biotechnology, Inc. (Santa Cruz, CA). Densitometric analyses were performed with ImageJ software (National Institutes of Health, Bethesda, MD).

### Real time-polymerase chain reaction analysis

Total ribonucleic acids (RNAs) were extracted from keloid fibroblasts using TRIzol Reagent (Invitrogen, Carlsbad, CA). Complementary deoxyribonucleic acid (cDNA) templates were prepared using oligo(dT) random primers and Moloney Murine Leukemia Virus (MoMLV) reverse transcriptase. After the reverse transcription reaction, the cDNA template was amplified by polymerase chain reaction (PCR) using Taq polymerase (Invitrogen). *PARP1* was quantitated by real-time PCR (LightCycler; Roche Molecular Biochemicals, Mannheim, Germany) using SYBR Green as the fluorophore (Molecular Probes, Eugene, OR). Primers of *PARP1* were as follows: forward: 5′-ggagtggatgaagtggcgaa-3′; and reverse: 5′-ggcgatcttggaccgagtag-3′. Glyceraldehyde-3-phosphate dehydrogenase (GAPDH) gene expression was used as a control. The level of *PARP1* mRNA expression was calculated as the relative intensity of the PCR product bands compared with that from the GAPDH gene using the 2^–ΔΔCt^ method. The mRNA expressions of TGF-β, and matrix metallopeptidases (MMPs) were also assessed. All PCR experiments were performed in triplicate.

### Statistical analyses

Statistical analyses were performed using PASW version 21.0 (SPSS, Inc., Chicago, IL). All experimental results were obtained from three independent experiments using cells from three separate isolations and are presented as mean ± standard deviation (SD). For comparisons between groups, data were analyzed by the Mann–Whitney U test or one-way ANOVA. For all tests, *p* < 0.05 was regarded as statistically significant.

## Results

### Patient demographics

A total of nine patients with eight keloids were achieved. Table 1 shows the baseline demographics of patients included in this study. All patients were female with mean age of 31.2. Six patients had skin type F I-II and other three patients had F III-IV. The duration of scar ranged between 2–30 years. The fibroblasts were isolated from all patients' scar, and then used for all following *in vitro* analyses.

### The protein expressions of *PARP1* and smad3 were enhanced in keloid tissue as compared with normal dermal tissue

**Figure 1.**
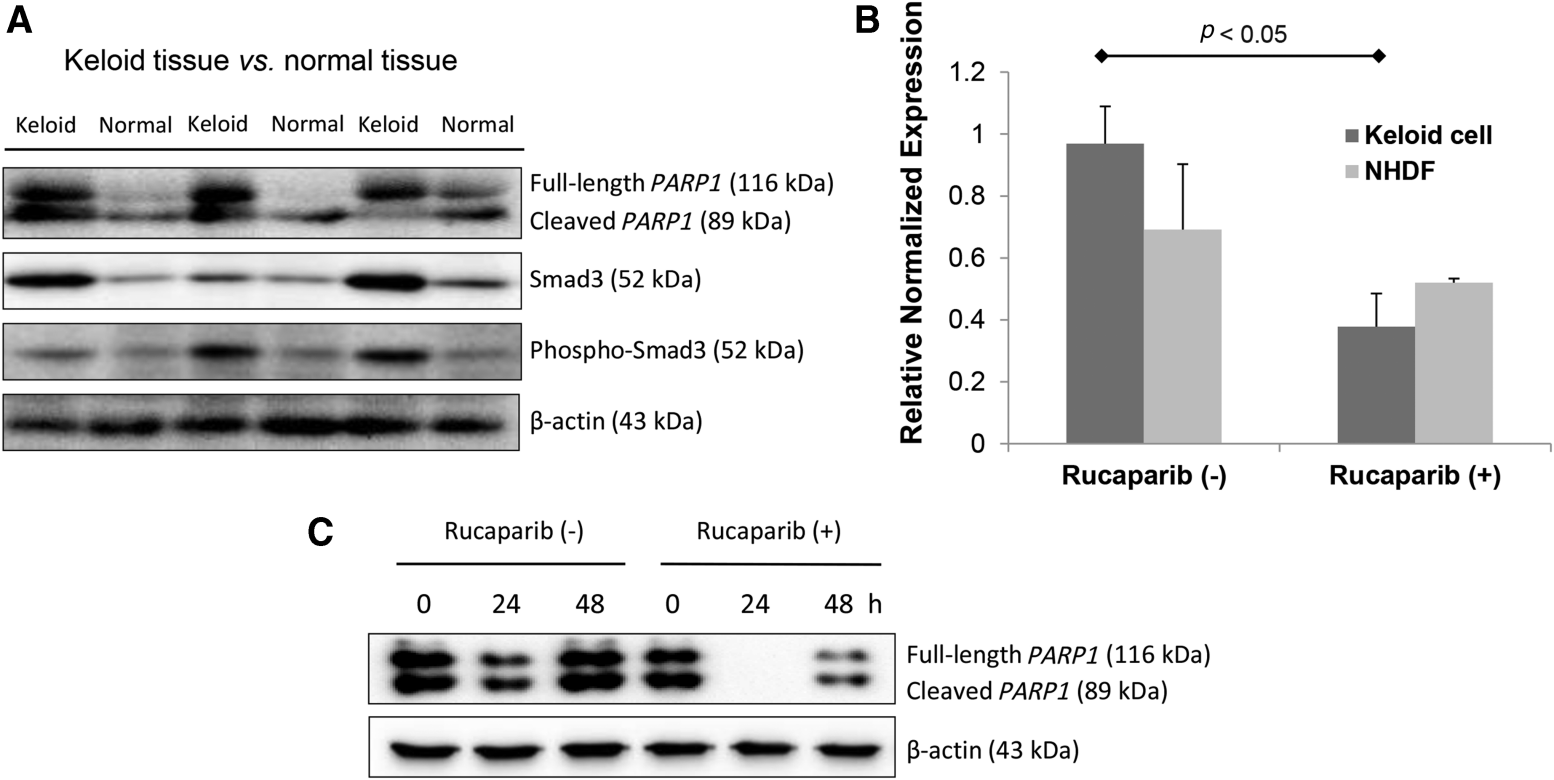
Rucaparib inhibited the expression of *PARP1* in keloid fibroblasts. **(A)** Immunoblot analyses revealed that the protein expressions of *PARP1*, smad3, and phosphorylated-smad3 were enhanced in keloid tissue as compared with normal dermal tissue. **(B)** Real-time PCR revealed that rucaparib treatment (20 μM) significantly decreased *PARP1* mRNA expression on keloid fibroblasts normalized to GAPDH expression levels. The experiments were repeated nine times. **(C)** Immunoblot analyses revealed that *PARP1* protein expression was suppressed by rucaparib treatment (20 μM). GAPDH, glyceraldehyde-3-phosphate dehydrogenase; NHDF, normal human dermal fibroblasts; *PARP1*, poly(ADP-ribose) polymerase 1.

First, we evaluated the protein expressions of *PARP1*, smad3, and phosphorylated smad3 in keloid tissue, which are the key signaling pathways in forming a keloid. As shown in Figure 1A, the protein expressions of *PARP1*, smad3, and phosphorylated smad3 were significantly enhanced in keloid tissue as compared with normal dermal tissue.

### The effect of rucaparib on the expression of *PARP1* in keloid fibroblasts

The expression of *PARP1* mRNA on keloid cell was significantly suppressed by rucaparib treatment (20 μM), whereas that on NHDF was not suppressed by rucaparib treatment (Fig. 1B; *p* < 0.05). We also analyzed the effect of rucaparib on the expression *PARP1* protein. Rucaparib treatment (20 μM) significantly attenuated the expression of *PARP1* protein as shown in Figure 1C.

### The therapeutic efficacy of rucaparib in keloid fibroblasts

**Figure 2.**
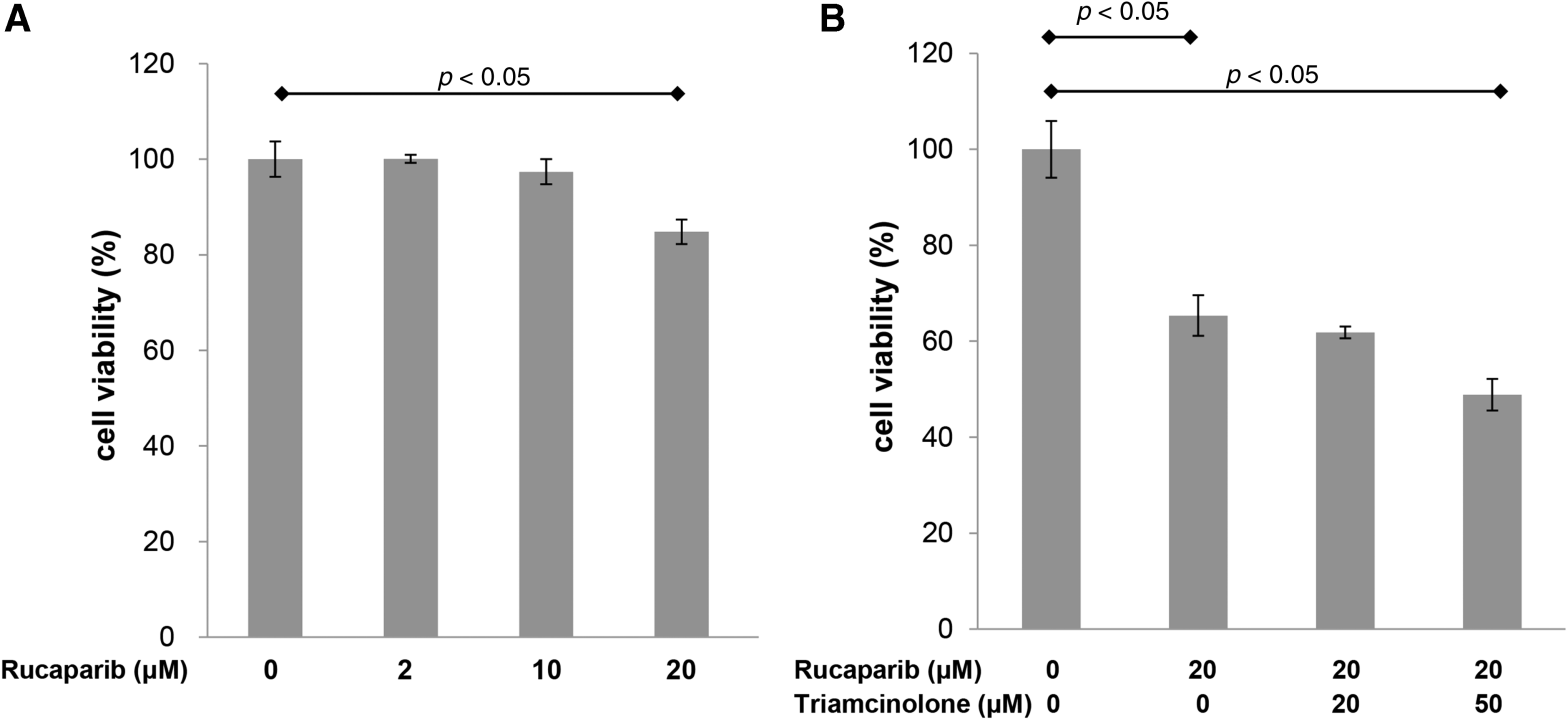
The effect of rucaparib on proliferation of keloid fibroblasts. **(A)** Keloid cell growth following treatment with rucaparib at various concentration (0, 2, 10, 20 μM) was evaluated by MTT assays. Data are expression as mean standard deviation of percent changes of triplicate optical densities. **(B)** Rucaparib (20 μM) significantly decreased proliferation of keloid fibroblasts. The combination of rucaparib (20 μM) and triamcinolone (50 μM) showed additive suppressive effect on keloid fibroblasts as compared with rucaparib single therapy. Data are expression as mean standard deviation of percent changes of triplicate optical densities. MTT, 3-(4,5-dimethylthiazol-2-yl)-2,5-diphenyltetrazolium bromide.

Then, we evaluated whether rucaparib ± triamcinolone suppress proliferation of keloid fibroblasts. Keloid cell growth following treatment with rucaparib at various concentrations (0, 2, 10, 20 μM) was evaluated by MTT assays. As shown in Figure 2A, rucaparib (20 μM) significantly suppressed the proliferation of keloid fibroblasts. Moreover, the combination of rucaparib (20 μM) and triamcinolone (50 μM) showed additive suppressive effect on keloid fibroblasts as compared with rucaparib single therapy (Fig. 2B).

### Rucaparib significantly decreased migration of keloid fibroblasts

**Figure 3.**
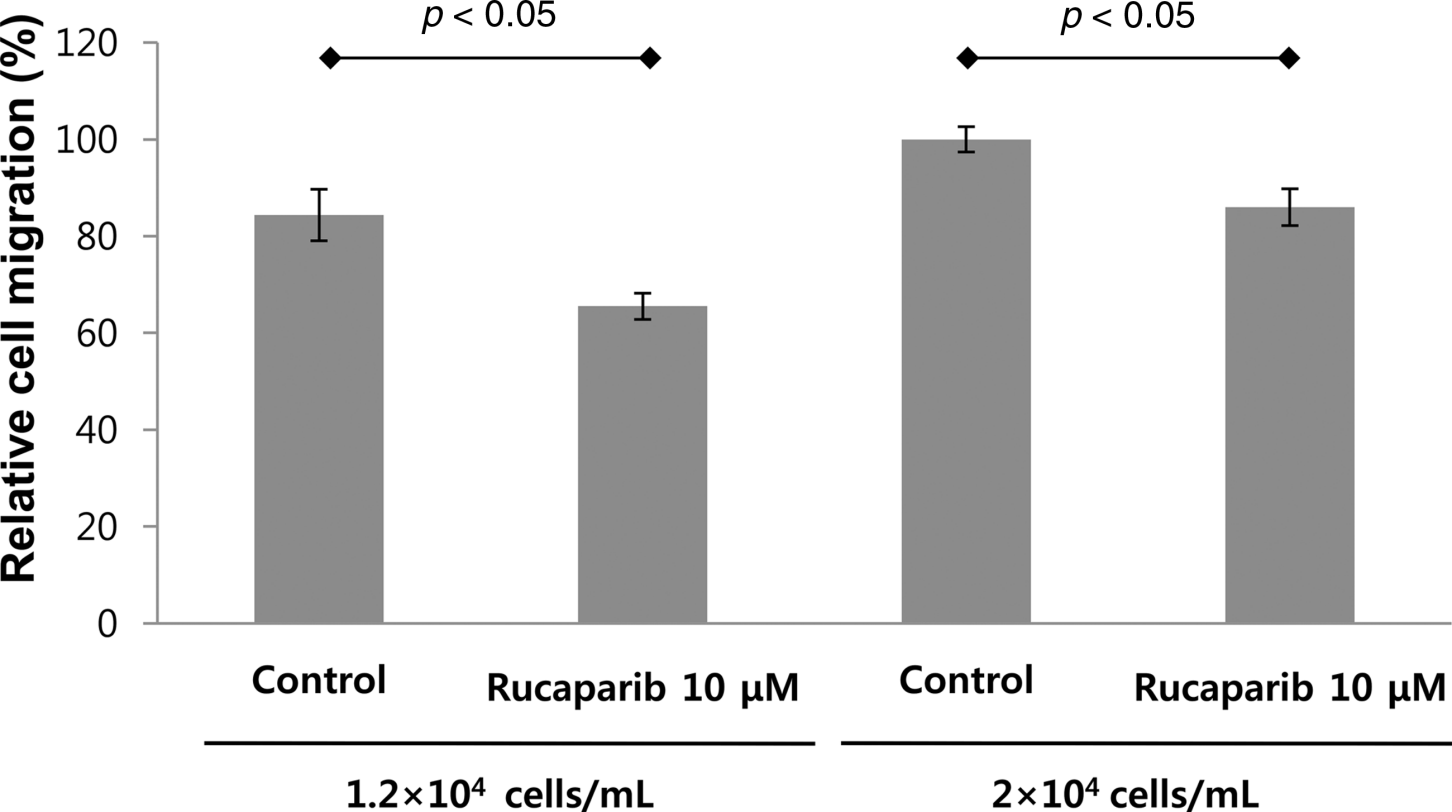
Rucaparib suppressed migration of keloid fibroblasts. The migration activity of dermal fibroblasts was decreased following rucaparib treatment (10 μM). The migration of the cells was analyzed by the transwell assay.

We compared the migration ability of keloid fibroblasts using the transwell assay. In comparison with the untreated fibroblasts, those treated with rucaparib (10 μM; the concentration that does not significantly suppress the proliferation of keloid cells) showed attenuated migration ability in both concentrations of 1.2 × 10^4^ and 2 × 10^4^ cells/mL (Fig. 3).

### Rucaparib significantly suppressed the fibrosis markers in keloid fibroblasts

**Figure 4.**
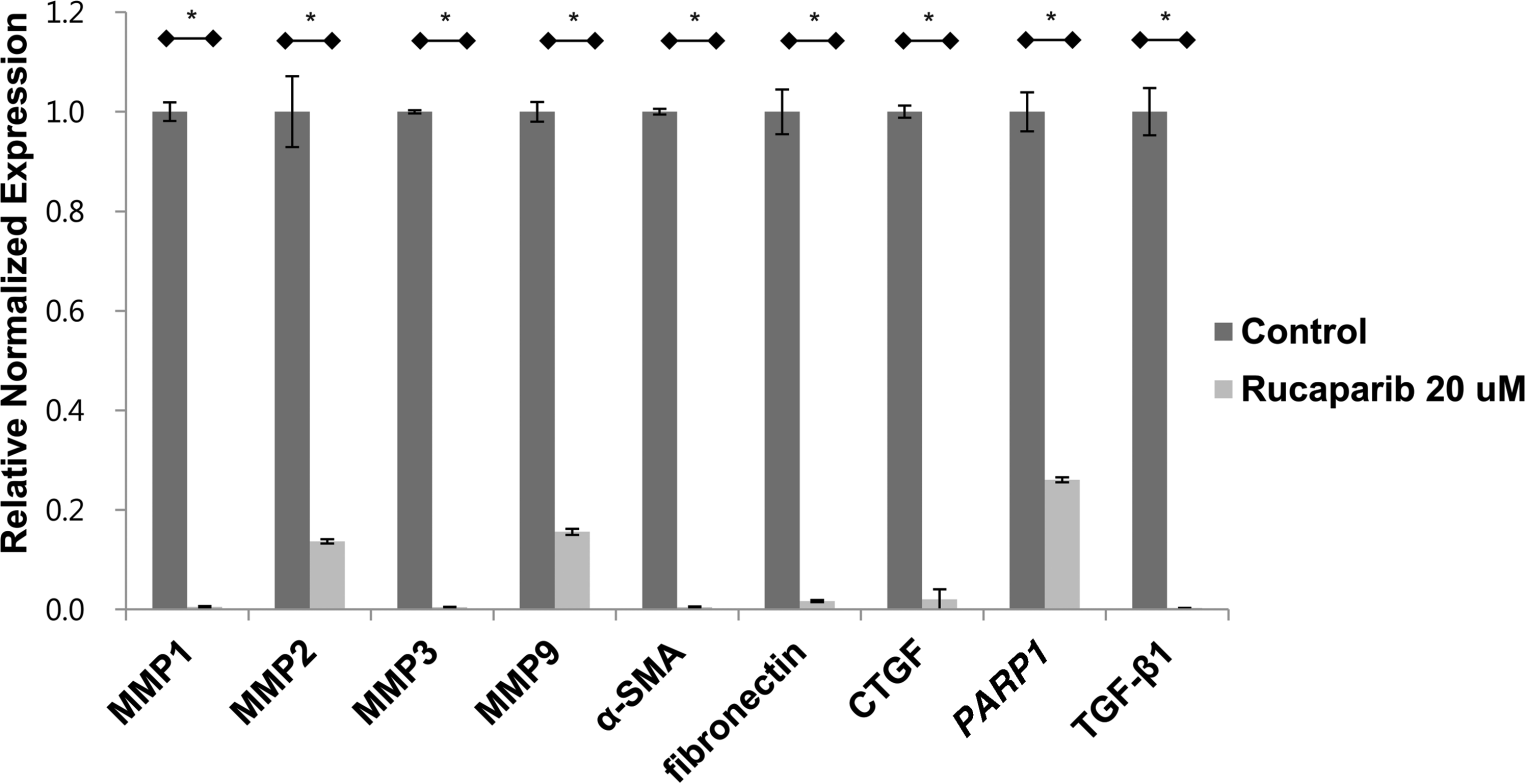
Rucaparib attenuated the expression of fibrosis markers in keloid fibroblasts. Real-time PCR revealed that rucaparib treatment (20 μM) significantly decreased the mRNA expressions of MMP-1, 2, 3, 9, and α-SMA, fibronectin, and CTGF on keloid fibroblasts normalized to GAPDH expression levels. *p < 0.05. CTGF, connective tissue growth factor; MMP, matrix metallopeptidase; PCR, polymerase chain reaction; SMA, smooth muscle actin; TGF-β1, transforming growth factor-beta 1.

We finally evaluated whether rucaparib reduces the expression of fibrosis markers in keloid cells. The mRNA expression of fibrosis markers, including MMP-1, -2, -3, and -9, α-smooth muscle actin, fibronectin, and connective tissue growth factor was significantly attenuated in keloid fibroblasts after rucaparib treatment (20 μM) (Fig. 4)

### Rucaparib significantly suppressed the fibrosis markers in keloid fibroblasts

**Figure 5.**
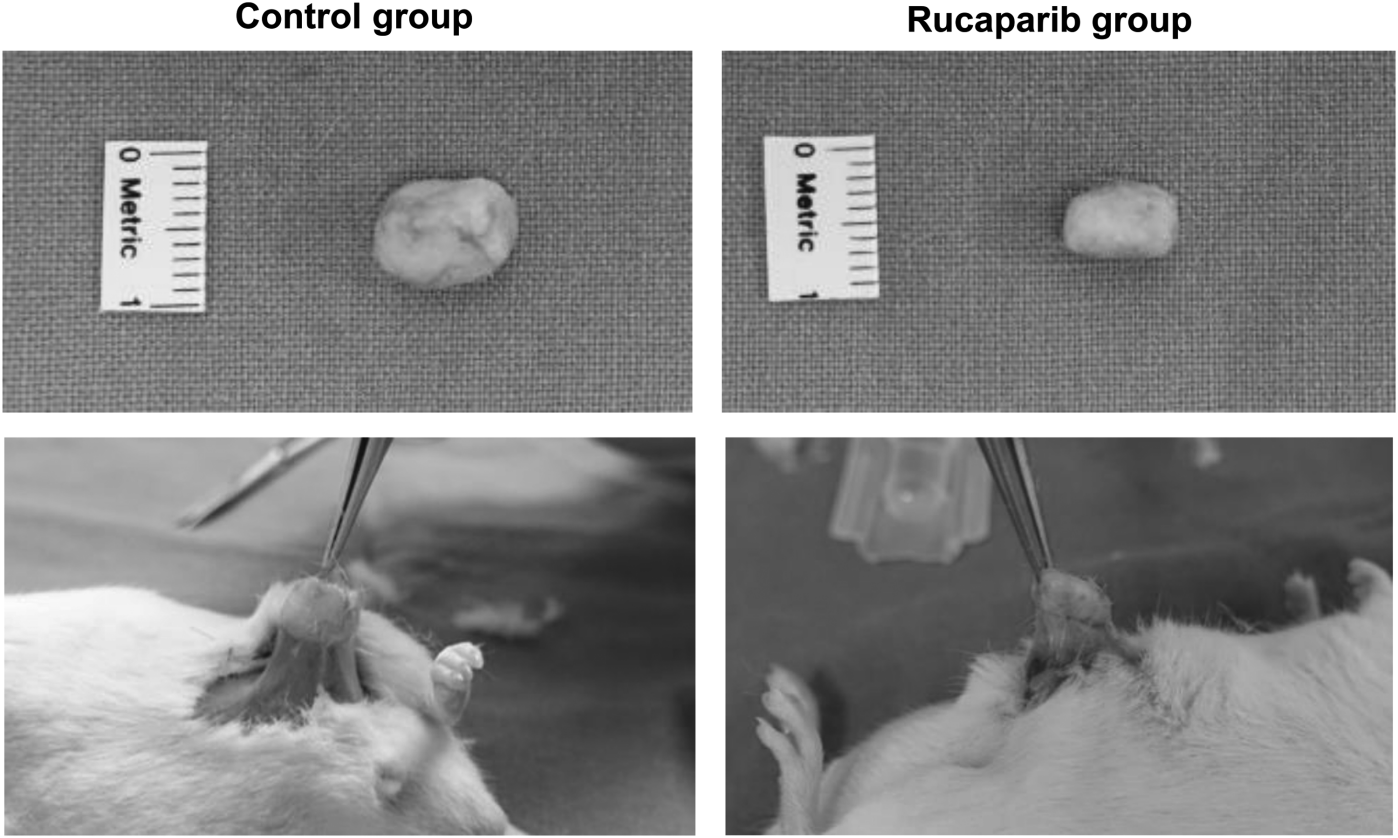
Rucaparib decreased the dimension of implanted keloid tissue in keloid xenograft model. Rucaparib significantly reduced the dimension of patient-derived implanted keloid tissue after 12 weeks as compared with control.

We evaluated whether rucaparib reduces the fibrosis in keloid xenograft model. According to two-dimensional measurements, rucaparib significantly reduced its size along with relative softness compared with the control group (Fig. 5). The relative size of explanted keloid tissue in the experimental group is 55% compared with the control group.

### Rucaparib significantly reduced the dimension of keloid tissue in patient-derived keloid xenograft model

We implanted the same-sized deepithelialized human keloid tissue (1 × 1 × 1 cm) into the subcutaneous pocket of 7 weeks old ICR mice (*n* = 4), and closed the wound. After 7 days, rucaparib (1 mM, 0.5 mL) or PBS (0.5 mL) was injected subdermally just around the implanted site. At the time of postoperative 12 weeks, the mean dimension of keloid tissue of rucaparib group was 0.9 × 0.5 cm, whereas that of control group was 1 × 1 cm (Fig. 5). Moreover, the implanted tissue in rucaparib group had much less adhesion with subcutaneous pocket compared with that in control group. We also measured the degree of firmness of the mass manually, which revealed five out of five in the control group, whereas two out of five in the rucaparib group.

## Discussion

Keloids, which occur because of unbalanced homeostasis in normal wound healing, do not regress spontaneously and frequently recur after surgical excision alone. The development of keloid scars involves a complicated multistep process that is likely driven by many different genetic, environmental, and local mechanophysiological factors. To date, no theory of the general mechanisms of pathologic scar formation has provided a full explanation of the clinicopathological characteristics of keloid.

*PARP-1* is known to affect the transcriptional output of a Smad-sensitive promoter, which suggests that *PARP-1* may play a more fundamental role in negatively regulating expression of TGF-β-responsive genes.^28^ Functionally, *PARP-1* is known to regulate transcription factors, such as p53, Sp1, and NF-κB.^29^ In addition, p53,^30^ Sp1,^31^ and NF-kB^32^ is also well known as one of the factors involved in keloid pathogenesis. In addition, *PARP1* is known to prerequisite for TGF-β1-induced Smad3 activation in rat vascular smooth muscle cells. These series of molecular findings implies that targeting *PARP1* may be a promising therapeutic target against keloid diseases characterized by dysregulated TGF-β/Smad3 pathway.

On the other hand, *PARP1* has been in the recent spotlight due to its strong relation with fibrotic disease in various organs. In cardiac tissue, *PARP-1* inhibition partially decreased autophagy, abrogated cardiac fibrosis, and significantly improved cardiac function post-MI.^33^ In renal tissue, PARP enzyme inhibition alleviates diabetic nephropathy through decreasing inflammation, oxidative stress, and renal fibrosis in diabetic animals.^34^ In liver, PARP inhibition protects against alcoholic and nonalcoholic steatohepatitis by attenuating hepatic triglyceride accumulation, metabolic dysregulation, or inflammation and/or fibrosis in models of nonalcoholic steatohepatitis.^35^ *PARP1* loss or downregulation itself alters the expression of many genes involved in cell cycle control and stress response, especially p53.^22^

Despite recent progress in recognizing its important role of PARP activation in fibrotic disease, however, no study unveiling its possible role in skin fibrosis, including keloid disease, has yet been done.

So, we initiated this study by analyzing baseline *PARP1* expression in normal human dermal tissue and keloid tissue. To our expectation, *PARP1* was highly expressed in keloid tissue compared with normal human dermal tissue. We also revealed that *PARP1* inhibition significantly inhibits the expression of keloid fibroblast in terms of cell viability, cell migration, and fibrosis marker expression. We performed an *in vivo* study to increase clinical significance and successfully demonstrated that *PARP1* inhibitor rucaparib, the FDA-approved chemotherapeutic drug for ovarian cancer, significantly decreased the degree of fibrosis in patient-derived keloid xenograft model. Conclusively, these findings suggested that *PARP1* inhibition can be a potential therapeutic target for keloid treatment and insinuated rucaparib can be a successful option for the treatment of keloid disease.

We also investigated TGF-β expression according to the delivered drugs. Even though both TA and *PARP1* inhibitors significantly decreased TGF-β expression, *PARP1* inhibitors showed a sharper decrease revealing an intimate relationship between *PARP1* and TGF-β. In addition, we think that *PARP1* inhibitors can be added to traditional TA to increase therapeutic efficacy based on our study results. However, this concept should be further studied in a clinical setting to optimize dose to be delivered and its composition.

The *PARP1* inhibitor, rucaparib, is a recently FDA-approved therapeutic agent for ovarian cancer. This is the first study reporting that PARP is overexpressed in keloid disease and that pharmacological inhibition with rucaparib significantly attenuates the expression of fibrosis markers *in vitro* and *in vivo*. Altogether, *PARP1* inhibition might be a promising therapeutic strategy for the treatment of keloid disease.

## Innovation

The study data suggest that *PARP1* might be a novel therapeutic target for keloid disease. *PARP1* inhibitor, rucaparib, might be a promising therapeutic drug for the treatment of keloid disease.

Key Findings

- The expressions of *PARP1* and Smad3 were significantly higher in keloid tissue.
- Rucaparib significantly suppressed the migration of keloid fibroblasts.
- Rucaparib significantly suppressed the fibrosis markers of keloid fibroblasts.
- The combination of rucaparib and triamcinolone showed additive suppressive effect.
- Rucaparib significantly reduced the size of keloid tissue *in vivo*.

## About the Authors

**Tae Hwan Park, MD, PhD** is working as an assistant professor at the Department of Plastic and Reconstructive surgery, CHA Bundang Medical Center, Korea. He is currently affiliated with Georgetown University, Washington D.C. as a visiting scholar. **Yuri Cho, MD, PhD**, co-corresponding author of this article, is working as an assistant professor at the Department of Internal Medicine, CHA Gangnam Medical Center, Korea. She has interests in cancer treatment and neoplastic diseases. **Chan Woo Kim, MD** is a chief resident working with Dr. Park TH. **Jin Sik Choi, MD**, who is a research associate with Dr. Park TH, and **Yun Joo Park, MD**, who is working as an assistant professor at the Department of Radiology in Hallym University Sacred Heart Hospital, Korea, have collaborated this project with Dr. Park TH. **Yosep Chong, MD**, who is working as an assistant professor at the Department of Pathology in Catholic University, St. Mary's Hospital, Korea, have collaborated this project with Dr. Park TH **Min Ji Park, MSc**, who is a full-time researcher working at CHA Gangnam Medical Center with Dr. Cho Y.

## Abbreviations Used

CTGF: connective tissue growth factor
DMEM: Dulbecco's modified Eagle's medium
FBS: fetal bovine serum
GAPDH: glyceraldehyde-3-phosphate dehydrogenase
MMP: matrix metallopeptidase
MTT: 3-(4,5-dimethylthiazol-2-yl)-2,5-diphenyltetrazolium bromide
NHDF: normal human dermal fibroblasts
*PARP1*: poly(ADP-ribose) polymerase 1
PCR: polymerase chain reaction
SD: standard deviation
SMA: smooth muscle actin
TGF-β1: transforming growth factor-beta 1
